# Cocaine, Appetitive Memory and Neural Connectivity

**DOI:** 10.4172/2161-0495.S7-003

**Published:** 2012-07-13

**Authors:** Suchismita Ray

**Affiliations:** Center of Alcohol Studies, Rutgers, The State University of New Jersey, 607 Allison Road, Piscataway, New Jersey, USA

**Keywords:** Appetitive memory, Neural connectivity, Cocaine addiction

## Abstract

This review examines existing cognitive experimental and brain imaging research related to cocaine addiction. In section 1, previous studies that have examined cognitive processes, such as implicit and explicit memory processes in cocaine users are reported. Next, in section 2, brain imaging studies are reported that have used chronic users of cocaine as study participants. In section 3, several conclusions are drawn. They are: (a) in cognitive experimental literature, no study has examined both implicit and explicit memory processes involving cocaine related visual information in the same cocaine user, (b) neural mechanisms underlying implicit and explicit memory processes for cocaine-related visual cues have not been directly investigated in cocaine users in the imaging literature, and (c) none of the previous imaging studies has examined connectivity between the memory system and craving system in the brain of chronic users of cocaine. Finally, future directions in the field of cocaine addiction are suggested.

## Introduction

According to the 2007 National Survey on Drug Use and Health, approximately 35.9 million Americans aged 12 and older had tried cocaine at least once in their lifetimes, representing 14.5% of this population. Among students surveyed as part of the 2008 Monitoring the Future study, 3.0% of eighth graders, 4.5% of tenth graders, and 7.2% of twelfth graders reported lifetime use of cocaine. Approximately 8.5% of college students and 14.7% of young adults (aged 19–28) reported lifetime use of cocaine (NIDA, 2007). Of an estimated 113 million emergency department (ED) visits in the U.S. during 2006, the Drug Abuse Warning Network (DAWN) estimates 548,608 were cocaine related. Despite this widespread and continuing use of cocaine in youth and young adults, much remains unknown about the neurocognitive mechanisms that support initiation and persistence of chronic cocaine use in humans. Better understanding of these mechanisms is essential both for identifying those at risk for cocaine use and for intervention development in persistent cocaine users. Motivated by this fact, I have included three sections in this review. First, I examine previous studies that have investigated cognitive processes, for example, implicit and explicit memory processes in cocaine abusers and cocaine-dependent individuals (section I). Next, I report brain imaging studies related to cocaine addiction (section II), and finally I draw conclusions from previous cognitive and imaging studies and recommend future directions (section III).

## Implicit and explicit memory processes and cocaine addiction

A long-standing cognitive research tradition has distinguished between automatic and non-automatic modes of information processing [1–8]. The automatic (i.e., implicit) mode involves faster, less effortful processing with less reliance on attention, intention, and strategy. It may occur without conscious awareness and may be difficult to control or inhibit once initiated. Implicit memory processes appear to play a crucial role in the control of attention and contingency-guided decision-making. On the other hand, the controlled and non-automatic (i.e., explicit) mode involves slower, more effortful processing such as rehearsal and elaboration and is dependent on strategy and attentional resources. Controlled information processes are initiated intentionally and are influenced by encoding strategies or depth of encoding [9]. Because explicit memory processes are supported by attentional and other cognitive resources that are limited in capacity, they are vulnerable to a variety of alterations in physiological, neurological, and psychological state [10,11]. Although prominent cognitive and neuroadaptive theories of addiction point to the heuristic value of the automatic/non-automatic information processing distinction for understanding the development of problem drug use behaviors [12–14], literature is however limited on systematic studies of specific types of automatic and effortful memory processes in chronic users of cocaine.

Lambert et al. [15,16] demonstrated that implicitly learned contingencies between cue and target stimuli, of which participants were unaware, guided attention-orienting responses. Their results support the idea that attention orienting to drugs and drug-related stimuli can operate outside of voluntary control via implicit memory processes. Bargh’s model [17] suggests that the environment can directly activate a goal (for example, ‘want to feel high’) that guides cognitive and behavioral processes without the need for conscious decision making, and without the person’s conscious intent or awareness of the operation of the goal [18]. The role of associative learning and implicit memory systems in contributing to transitions from regular drug use to addiction [12] and to the function of craving [19] is well supported, yet little attention has been directed to the role of implicit memory as an explanatory mechanism for early transitions in use, such as experimental use to occasional use, and occasional use to regular and/or escalating use. Exceptions are the earlier work by Stacy et al. [20,21], Szalay et al. [22–24], and Hill and Paynter [25], who examined motivational processes using verbal methods of assessing memory activation. Stacy [20], for example, showed that memory associations to ambiguous cues (e.g., ‘pitcher’) were significantly related to college students’ use of alcohol and marijuana use. Furthermore, Szalay et al. [22–24] research showed that college cocaine users associate cocaine words with positive experiences and feelings, and are more familiar with cocaine names than college student nonusers. Hill and Paynter [25] used an implicit semantic priming paradigm that included appetitive cues and suggested that this paradigm may have value as a clinical tool for the detection of psychoactive substance dependence and assessment of change. Semantic priming is defined as recognition facilitation of semantically related stimuli. Hill and Paynter [25] found that lexical decision (LD; the time to decide whether a string of letters was a word or a non-word) time was faster for the alcohol-related words [e.g., ‘alcohol’ (study word)–’relaxation’ (test word)] than unrelated words [‘alcohol’ (study word)–’navigation’ (test word)]. This suggests stronger memory association for alcohol-related concepts in semantic memory network than between concepts that are not alcohol related.

In the opiate addiction literature, only one study has examined contextual priming in opiate-dependent individuals and their family members and found that dependent individuals responded faster to opiate-related words following withdrawal-related sentences, as compared to neutral words that followed neutral sentences [26]. In the cocaine addiction literature, there is one study that has examined implicit and explicit cocaine-related cognitions in cocaine-dependent poly-substance abusers and controls [27]; implicit associations were assessed using an implicit association test (IAT) and explicit cognitions were assessed with a questionnaire using the same words as the IAT. Results showed that cocaine patients, compared to controls, associated cocaine more strongly with arousal as measured in the IAT, and scored lower on sedation expectancies and higher on arousal expectancies as measured in the explicit test. In addition to the Wiers et al. and Szalay et al. [22–24,27] studies that have examined cocaine related memory association in cocaine users, there has been one previous study that has examined attentional bias for cocaine-related words in cocaine abusers [28] and another study that has examined visual neutral word priming in abstinent cocaine and cocaine/alcohol dependent volunteers [29]. In Franken et al. [28] study, cocaine abusers participated in a reaction time (RT) experiment that was intended to measure the ability of subjects to shift their attention away from the cocaine related words. Results showed attentional bias for cocaine cues in patients who scored higher on obsessive thoughts about cocaine use in the week before the experiment. According to Jasiukaitis and Fein [29] both semantically and perceptually mediated visual neutral word priming are based on implicit cognitive processes that are resilient to the sequelae of cocaine dependence. In contrast to enhanced cocaine-related implicit memory processing in cocaine users [22–24,27], explicit memory involving neutral stimuli is usually impaired in chronic heavy users of alcohol and other drugs [29–31]. Cocaine dependent individuals, as well as abstinent MDMA (‘Ecstasy’) users, show impaired explicit memory processes such as free recall that utilizes neutral stimuli [29,32]. From the above review, it is evident that none of the existing studies involving cocaine has examined both implicit and explicit memory processes for cocaine related visual information within the same cocaine abuser or cocaine dependent individual.

Drug use behaviors in chronic users are frequently triggered by viewing drug related visual cues in the environment. Investigating the integrated operation of implicit and explicit memory processes involving drug related visual information within the same individual has important implications for developing prevention and intervention techniques tailored specifically for that individual. Many drug abuse prevention interventions involve techniques such as drug refusal skill training, learning the negative consequences of drug use, and other controlled and attention-demanding components that rely heavily on explicit memory [33].

## Brain imaging studies related to cocaine addiction

The neural correlates of implicit and explicit memory processes have been well studied in healthy non-substance abusing individuals. Using implicit repetition priming and semantic priming paradigms and functional Magnetic Resonance Imaging (fMRI), the role of Left Dorsal Prefrontal Cortex (LDPC), Extrastriate Visual Cortex (EVC), and Posterior Temporal Cortex (PTC) has been demonstrated in implicit processing of neutral picture and word stimuli [34–42]. Priming is characterized by decreased brain activation (“response suppression”) in LDPC, EVC, and PTC for repeated compared to novel items (repetition priming) or for related compared to unrelated items (semantic priming). On the other hand, using explicit episodic and recognition memory tasks and fMRI, involvement of amygdala in explicit processing of emotional picture and word stimuli and involvement of hippocampus in explicit processing of neutral picture and word stimuli has been demonstrated [43–47]. Explicit episodic and recognition memory is characterized by increased activation in amygdala and hippocampus for correct recognition of the studied items compared to the novel items.

Neural mechanisms underlying implicit and explicit memory processes for appetitive cues have not been directly investigated in cocaine dependent individuals in the fMRI and PET literature. Yet, independent lines of research suggest that it would be valuable to do so. For example, cocaine-related cues and active cocaine infusion cause activation in brain areas linked to craving. Typically in a cue exposure paradigm, individuals maintaining abstinence from drug/alcohol use are brain scanned during exposure to addiction-related word or picture stimuli or related thoughts. Using cocaine-related verbal and picture cues, activation of insula, Orbito Frontal Cortex (OFC), amygdala, hippocampus and Anterior Cingulate (AC) areas was found during cocaine-cue induced craving [48–58]. These same brain areas have shown activation in response to alcohol, heroin, and nicotine-related cues in individuals dependent on these substances [59–68]. Cocaine-challenge studies showed that acute cocaine administration activated mesolimbic and mesocortical dopaminergic projection regions in addition to activation in anterior prefrontal cortex and orbitofrontal cortex [69–71]. These studies concluded that dopaminergic pathways and hierarchical brain networks may participate in mediating cocaine reward processes, associative learning, memory and motivation. Thus, drug and alcohol cue exposure is believed to trigger memories related to their use [28,49,51,65,72,73], apparently due to activation in hippocampus and amygdala during cue exposure. A separate line of research in healthy non-substance abusing individuals has linked activation in the hippocampus to explicit memory [44–47] and activation in the amygdala to both explicit emotional [43,44] and implicit emotional memory [74].

Taken together, the above imaging literature suggests a role of LDPC, EVC, and PTC in implicit processing of neutral stimuli, and involvement of amygdala and hippocampus in explicit processing of emotional and neutral stimuli, and also the role of insula, OFC, amygdala, hippocampus and AC areas in cue induced brain activation in cocaine and other substance use disordered individuals. In addition to examining localized brain Regions of Interest (ROIs), there has been an increasing focus of neuroscientists on understanding how one brain area influences another, that is, on the ‘effective connectivity’ [75] or ‘functional connectivity’ (according to this view, functional connectivity between two brain areas is the correlation or covariance between their time-dependent activity) [75] during a cognitive task or at rest in healthy normal and clinical and neurotypical individuals [76–94]. Functional connectivity provides a quantitative description of inter-relatedness of information processing in different regions of brain, pertaining to a certain cognitive task or in a resting state. In that sense, functional connectivity provides information about information flows in the brain and influences produced by different areas of the brain on each other during particular cognitive tasks. This typically provides correlative information but can be used to obtain hints about the causal links (from such correlations) that exist between different cognitive processes in the brain in subjects’ performing certain tasks. The modeling of effective connectivity not only provides an *in vivo* examination of brain function that complements the more invasive techniques used in animal research, but also has proved to be a useful tool for understanding brain function in both clinical and neurotypical populations. This modeling has been instrumental in developing clinical interventions. This approach expands the utility of neuroimaging data not only to identify isolated brain ROIs that are especially active during cognition, perception and action, but also the causal relations among activity in these regions [74,95–99]. The inter-connectivity of different regions in the brain is a well established concept in clinical and neurotypical literature.

However, this issue of brain interconnectivity is relatively novel in the addiction literature. Only one recent fMRI study has examined brain functional connectivity during a finger tapping task in MDMA abusers [100] whereas in another fMRI study brain effective connectivity was examined while the healthy subjects were under the influence of pure compounds of cannabis sativa. Furthermore, only one previous PET study of opiate craving [61] and only one previous resting state fMRI study involving opioid dependent patients [101] have investigated this important issue of brain interconnectivity. A few MRI studies that have examined functional connectivity in heroin abusers or heroin dependent individuals have primarily concentrated on the resting state [102–110] and functional or effective connectivity during any cognitive task yet needs to be explored. Similarly, the brain interconnectivity literature on cocaine addiction is very limited: only one study has examined the effect of acute cocaine administration on functional connectivity in human primary visual and motor cortex [111], a few studies have examined functional connectivity during the resting state [58,112–115] and a few studies have examined functional connectivity while participants performed a cognitive task [116–119].

## Conclusions and Future Directions

Review of the previous cognitive experimental literature involving cocaine addiction reveals that only a limited number of studies have examined cocaine-related implicit and explicit memory processing [23–27] in cocaine users, despite the fact that neurobiological models of addiction argue that memory processes, and especially implicit memory processes play a crucial role in escalation and persistence of drug abuse [12–14]. However, there is no study that examined both implicit and explicit memory processes involving cocaine related visual information in the same cocaine abuser or cocaine dependent individual. Future research is needed to examine this. Understanding that an individual’s explicit memory processes involving drug related visual information are impaired whereas the implicit memory processes involving the same information are spared will help develop prevention and intervention tools that will utilize that person’s intact implicit memory system. The implicit memory processes have not been the focus in the field, although it has been suggested that changing automatic associative effects could be fundamental adjunct to interventions [120].

Future work on the role of implicit memory in drug-taking behavior would be informed by studies on cognitive processes in the alcohol literature. In line with Goldman [121], it can be suggested that an implicit spread of activation between addiction related stimuli and their respective addiction expectancy concepts in the semantic memory network (a permanent associative network in which knowledge is stored) contributes to drug taking behavior. Goldman [121] has argued that alcohol expectancy concepts (images, memories of sensor motor and affective experiences, specific behavior patterns, and verbal representations of these concepts) are nodes in the semantic memory network and activation of particular nodes occurs in a predictable fashion once the individual encounters stimuli that match previously encoded material relevant to drinking. These activation patterns in turn influence the onset and pattern of drinking by activating affective systems in the brain [12]. It has been shown in the cognitive experimental literature that implicit memory processes are affected by divided attention [122] wherein one retains a high memory load during the memory processing. Future cognitive experimental research in the field of cocaine addiction should explore how attentional demands and memory loads might be harnessed or controlled for cocaine use prevention and intervention. That is, it may be possible to use attentional manipulations during exposure to a cocaine visual cue to restrict the implicit spreading of activation between, for example, cocaine related concepts in the semantic memory network.

In the fMRI and PET literature, neural mechanisms underlying implicit and explicit memory processes for cocaine cues have not been directly investigated either in high-risk for cocaine abuse/dependent or in cocaine dependent individuals despite an independent line of research has suggested that it would be valuable to do so. Furthermore, none of the previous imaging studies has examined connectivity between the memory system and craving system in the brain despite the fact that it has been argued that these two systems play a crucial role in maintenance of drug use behavior [12,19,123]. Future research should integrate the results of the neural substrate of memory in non-clinical samples with research examining the neural mechanisms of craving in substance use disordered groups by using fMRI to directly examine neural mechanisms that underlie implicit and explicit memory processing of cocaine-related visual cues in youthful samples who vary in cocaine exposure from none, to occasional, to persistent. Research should focus on brain ROIs that have shown to be involved in implicit memory processing (LDPC, EVC, PTC) and explicit memory processing (amygdala, hippocampus) in non-clinical samples, and brain ROIs that have shown to be involved during cocaine as well as other cue exposure in cocaine and other substance use disordered individuals (insula, OFC, amygdala, hippocampus, AC). Activation in LDPC, EVC, and PTC ROIs should be examined while participants’ process cocaine related visual information during an implicit memory task whereas activation in amygdala and hippocampus ROIs should be examined while they process cocaine related visual information during an explicit memory task. Also, during both memory tasks, activation of craving related brain areas (insula, OFC, AC) should be examined. In addition to examining the localized brain ROIs, it is necessary to further examine the connectivity between craving related and implicit (or explicit) memory related brain areas while participants process cocaine related visual cues during implicit (or explicit) memory task by using multiple advanced state-of-the art causal modeling approaches [75,124–126]. The research findings will elucidate our understanding of how brain functioning may differ in persons who vary in extent and consequences of cocaine exposure, that is, individuals who have limited experience with cocaine versus individuals who are chronic cocaine users. More specifically, in the high-risk group, the connectivity from the memory system to the craving system is not expected to be as strong as in the cocaine dependent individuals as strength of connectivity is a function of repeated exposure over time. Individuals with no previous experience with cocaine would not show any connectivity between these systems. Thus, the proposed research has practical implications in terms of its ability to assess an individual’s level of cocaine experience by examining that individual’s connectivity map which reveals the individual’s strength of connectivity between the memories and craving systems. Such knowledge will be useful in identifying individual specific treatments or drug targets.

Furthermore, an examination of the connectivity map between the memory and craving systems during the course of treatment may help clinicians identify individuals who may relapse. The novel medication development strategies for long-term smoked cocaine use [e.g., dopamine receptor agonist (modafinil), dopamine receptor antagonist (ecopipam)] should be examined on cocaine dependent individuals to see whether their subjective craving ratings change and whether the connectivity between the memory and craving systems is diminished as a result of medication. At a cognitive level, this will refer to a restriction on implicit spreading of activation between cocaine related concepts in the semantic memory network. Finally, knowledge gained from this research on neurocognitive mechanisms in cocaine addiction will be instrumental in developing therapies that will modulate the functions of craving related brain areas in cocaine dependent individuals.

In addition to the proposed above mentioned cognitive experimental and fMRI research, advances in structural integrity/connectivity obtained by utilizing both Diffusion Tensor Imaging (DTI) and Voxel Based Morphometry (VBM) imaging techniques [127,128] can enhance our understanding of brain dysfunction in cocaine dependent individuals. As these evolving methods mature, a better understanding of structural and functional connectivity and their interplay will further enhance the field.

The results from future research will lay the groundwork for more articulated neurocognitive models of craving and impulse control in cocaine users, and potentially suggest ways that implicit memory processes may be harnessed to interrupt craving states. The knowledge gained from this future research will have implications for developing individually-tailored and effective cocaine use prevention and intervention techniques. These techniques could potentially include cognitive restructuring within the implicit memory system, neuro feedback [129–132], developing therapies to modulate the functions of craving related brain areas, and medication development.
